# Bioprocessing strategies for the large-scale production of human mesenchymal stem cells: a review

**DOI:** 10.1186/s13287-015-0228-5

**Published:** 2015-11-23

**Authors:** Krishna M. Panchalingam, Sunghoon Jung, Lawrence Rosenberg, Leo A. Behie

**Affiliations:** Pharmaceutical Production Research Facility, Schulich School of Engineering, University of Calgary, 2500 University Drive NW, Calgary, AB T2N 1N4 Canada; Department of Surgery, McGill University Health Centre, 845 Rue Sherbrooke Quest, Montreal, QC H3G 1A4 Canada; Jewish General Hospital, 3755 Chemin de la Côte-Ste-Catherine Road, Montreal, QC H3T 1E2 Canada

## Abstract

Human mesenchymal stem cells (hMSCs), also called mesenchymal stromal cells, have been of great interest in regenerative medicine applications because of not only their differentiation potential but also their ability to secrete bioactive factors that can modulate the immune system and promote tissue repair. This potential has initiated many early-phase clinical studies for the treatment of various diseases, disorders, and injuries by using either hMSCs themselves or their secreted products. Currently, hMSCs for clinical use are generated through conventional static adherent cultures in the presence of fetal bovine serum or human-sourced supplements. However, these methods suffer from variable culture conditions (i.e., ill-defined medium components and heterogeneous culture environment) and thus are not ideal procedures to meet the expected future demand of quality-assured hMSCs for human therapeutic use. Optimizing a bioprocess to generate hMSCs or their secreted products (or both) promises to improve the efficacy as well as safety of this stem cell therapy. In this review, current media and methods for hMSC culture are outlined and bioprocess development strategies discussed.

## Introduction

Human mesenchymal stem cells (hMSCs) were first isolated from bone marrow but have since been found in other tissues in the body, such as adipose tissue, umbilical cord blood, the Wharton jelly of the umbilical cord, synovium, lung, pancreas, and muscle [1–3]. Whereas these other hMSC sources have emerged in the last few years and are being studied, bone marrow-derived hMSCs (BM-hMSCs) have been rigorously studied over many years and are used in the majority of hMSC clinical studies and trials. The clonogenic BM-hMSC fraction ranges from 10 to 100 CFU-F (colony-forming unit—fibroblast) per 10^6^ marrow mononuclear cells (MNCs) and is typically isolated and expanded in classic serum-based media on tissue culture plastic. BM-hMSCs are characterized by (a) their adherence to plastic; (b) multipotency (i.e., adipogenic, osteogenic, and chondrogenic differentiation); (c) positive expression of surface antigens CD73, CD90, and CD105; and (d) lack of CD34, CD45, CD14 or CD11b, CD19 or CD79α, and HLA-DR expression [4]. In addition to their multipotency, hMSCs have been shown to have the ability to secrete bioactive factors which can modulate the immune system (e.g., indoleamine 2,3-dioxygenase and prostaglandin E_2_) and promote tissue repair (e.g., glial cell line-derived neurotrophic factor and vascular endothelial growth factor, or VEGF) [5]. In fact, it is widely accepted that the majority of hMSC-mediated therapeutic benefits are due to their secretion of bioactive molecules as it has been shown that these factors have various therapeutic effects both in vitro and in vivo (i.e., anti-inflammatory, anti-fibrotic, anti-apoptotic, anti-angiogenic, or immunomodulatory) as well as repair/regenerative actions. To generate hMSCs for clinical studies, it is necessary to first expand these cells for several passages in vitro, after which adequate potency testing should be performed before cell infusion.

Any bioprocess used to produce therapeutic cells needs to be carefully designed, as this process is distinctly different from the well-known processes used to produce biopharmaceuticals. The first of these differences is that each batch or lot of therapeutic cells generated to treat one patient would be much smaller than the cell yields achieved for therapeutic protein production. Although hMSCs can be expanded for more than 40 population doublings (PDs) in culture, it has been suggested that cells of fewer than 20 PDs, particularly BM-hMSCs, be used for clinical applications with regard to safety and efficacy to avoid possible cell transformation [6, 7].

The second difference compared with therapeutic protein production is that hMSCs are the therapeutic product themselves. Thus, it is critical to produce functional hMSCs that retain their therapeutic properties. In this regard, it is important to develop a bioprocess for the expansion of hMSCs in a well-defined environment, where the nutritional, physiochemical, and mechanical requirements are met, controlled, and maintained (i.e., in bioreactors) for the culture period in order to generate consistent quantities of cells with the same desired properties. If variability is present between batches, this could undermine the therapeutic properties of the hMSCs. Hence, it is important to produce hMSCs for therapeutic applications in a well-defined manner (i.e., defined medium formulation) under good process control (i.e., online computer control in bioreactors) which can be operated in a closed system according to Good Manufacturing Practice (GMP).

## Human mesenchymal stem cell culture

### Culture media

Conventional medium used for isolating and expanding hMSCs is typically a defined basal medium—i.e., Dulbecco’s modified Eagle’s medium (DMEM)—supplemented with fetal bovine serum (FBS): 10–20 % (vol/vol). However, concerns exist with the use of FBS for clinical use: namely (a) the variability of FBS from batch to batch, (b) its ill-defined nature, and (c) the possibility that FBS contains harmful contaminants such as prions, viral, and zoonotic agents. Moreover, when hMSCs are cultured in a medium containing animal proteins, a substantial amount of these proteins is retained in the cytoplasm of hMSCs, which may elicit an immunologic reaction when the cells are transplanted in vivo [8]. It is for this reason that, even though FBS is still widely used in hMSC research, it has been suggested (by our group and others) that the development of a defined serum-free medium is needed for the expansion of quality-assured clinically acceptable hMSCs [9–11].

#### Humanized media

To find a suitable replacement for FBS, human blood-derived materials such as human serum and platelet derivatives have been investigated as an alternative medium supplement (reviewed in [10, 12]). Although human autologous serum has been reported to support hMSC expansion, it would be difficult to procure sufficient quantities of this serum to generate clinically relevant numbers of hMSCs [13–15]. Allogeneic human AB serum would circumvent this issue as several donor serums could be pooled to eliminate donor-specific differences and produced in a large-scale manner. Moreover, some groups have reported that it performs as well as FBS [16–18]. It has also been reported by many groups that human platelet lysate (hPL) or platelet-rich plasma have considerable growth-promoting properties for hMSCs while maintaining their differentiation potential and immunomodulatory properties [19–22]. However, one study reported that although hPL supported the expansion of hMSCs, it also impaired their immunomodulatory capacity [23]. Moreover, two other studies reported that a reduction in the osteogenic or adipogenic differentiation potential was seen in hPL-expanded hMSCs [24, 25]. Although these alternatives may be safer than using FBS and are currently being exploited for some clinical trials, the use of human-sourced supplements is still a matter of substantial debate, prompting concern in that there is still a risk that these supplements might be contaminated with human pathogens not detected by routine screening of blood donors. Moreover, these crude blood derivatives are poorly defined and can suffer from batch-to-batch variation (as reported for hPL in [26]), and thus their ability to maintain hMSC growth and therapeutic potentials could be widely variable. As it can be difficult to obtain reproducible and consistent cell quantities and qualities using these human sources, it can be a hindrance in the development of quality-assured hMSCs for large clinical studies. Therefore, effort should be made to standardize the production of these materials, limit the donor-to-donor variability (i.e., through pooling), and establish methods for pathogen inactivation [27, 28].

#### Defined serum-free media

There has been much progress in the last few years to develop serum-free media for the isolation and expansion of primary hMSCs (reviewed in [10, 29]). Although numerous commercial formulations have been released, our group reported in detail the first defined serum-free medium formulation (i.e., PPRF-msc6) which supported the rapid isolation and expansion of hMSCs from BM MNCs and their subsequent passages while maintaining their immunophenotype and multipotency [9, 30].

To the best of our knowledge, only two commercial serum-free media have been shown to support the isolation and expansion of primary hMSCs. Miwa et al. reported that they were able to isolate and expand hMSCs from BM MNCs in Mesencult-XF (Stemcell Technologies, Vancouver, BC, Canada) and in comparison with serum-supplemented culture observed higher cumulative PDs of 22–23 PDs in Mesencult-XF and 13–14 PDs in serum-supplemented cultures [31]. Moreover, Gottipamula et al. reported that they were able to isolate and expand hMSCs from BM MNCs in Becton Dickinson (Franklin Lakes, NJ, USA) Mosaic hMSC Serum-Free medium (BD-SF) and achieve cell yields comparable to that achieved for cells isolated and expanded with Mesencult-XF [10]. However, the formulations of these commercial media are not disclosed, and thus it may restrict their wide utility in hMSC research and clinical studies where the formulations cannot be exploited or modified.

### Culture mode

#### Adherent and spheroid culture

hMSCs are typically isolated and characterized by their adherence to plastic. However, the adherent culture of hMSCs may alter their phenotype and therapeutic properties as it represents an environment which is a different from their niche in vivo [32]. In fact, many observations have suggested that pre-conditioning hMSCs with either biological factors or the culture condition can enhance the therapeutic properties of hMSCs [33–36]. One method is the culture of hMSCs as spheroids (reviewed in [32]). In fact, Bartosh et al. found that the aggregation of hMSCs enhanced their anti-inflammatory properties, namely the increased expression of TSG-6 and stanniocalcin-1 [37]. Also, spheroid culture expressed high levels of three anticancer proteins: interleukin-24, tumor necrosis factor-alpha (TNF-α)-related apoptosis-inducing ligand, and CD82 [37]. Zimmermann and McDevitt also observed that the formation of hMSC aggregates can enhance the anti-inflammatory properties of the cells and that if the cells are treated with TNF-α and interferon-gamma they can inhibit the secretion of TNF-α by macrophages [38]. The benefit of spheroid culture has also been observed in pre-clinical studies where transplantation of hMSCs from adipose tissue into porcine models improved cell retention, survival, and integration [39, 40]. However, for this culture method to be applied in the clinic on a large scale, robust data on the growth kinetics and phenotype of the cells will need to be gathered. As Zimmermann and McDevitt noted, the immunomodulatory factor secretion was highly dependent on the composition of the cell culture medium [38]. And it may be necessary to re-develop a medium more suitable to the expansion of hMSCs as spheroids (rather than as adherent cells), as was done by Alimperti et al. [41].

#### Normoxic and hypoxic culture

The expansion of hMSCs in vitro has often been done at atmospheric oxygen levels of 21 %. It has been reported that exposure of hMSCs to these levels can induce DNA damage, contributing to cellular senescence and decreased therapeutic efficacy [42]. When culturing hMSCs at physiological oxygen levels (i.e., 1–5 %), an increase in cell growth and an increase in their adipogenic and osteogenic differentiation have been observed [43]. Additionally, hMSCs expanded at these low oxygen conditions have limited oxidative stress, DNA damage, telomere shortening, and chromosomal abnormalities [42]. Also, the exposure of hMSCs to low physiological oxygen levels can pre-condition them before transplantation and increase their therapeutic ability [44–47]. To mimic the ischemic microenvironment, serum-expanded hMSCs were placed in a serum-deprived medium under hypoxia and were found to secrete increased levels of pro-angiogenic factors that included VEGF-A, angiopoietins, insulin-like growth factor 1, and hepatocyte growth factor (HGF) [48]. Additionally, using an in vivo modified chick chorioallantoic membrane angiogenesis assay, the hypoxic-serum-deprived hMSCs displayed significantly higher angiogenic potential compared with typical culture-expanded hMSCs. Recently, Chang et al. showed that hypoxic preconditioning of BM-hMSCs and transplantation of this conditioned medium (CM) into rats with experimental traumatic brain injury (TBI) resulted in these rats performing significantly better in both motor and cognitive function tests as well as showing increased neurogenesis and decreased brain damage compared with TBI rats transplanted with CM collected from normoxic-expanded BM-MSCs [44]. Moreover, hypoxic conditions were able to stimulate the BM-hMSCs to secrete higher levels of VEGF and HGF. Therefore, given these observations, it may be necessary to consider expanding hMSCs in hypoxic conditions before transplanting the cells in vivo, in order to enhance their survival and therapeutic potential.

## Large-scale expansion

There are many types of bioreactors used for the expansion of hMSCs (reviewed in [49–52]). The most widely used bioreactors in the laboratory are tissue culture flasks, which provide a surface for hMSCs to adhere to and which are available with different surface areas: 25, 75, 150, and 225 cm^2^. These are cost-efficient and easy-to-operate and provide good gas exchange with the external environment through a filter cap or cracked plug cap and ample headspace. However, for the generation of a large number of hMSCs in clinical applications, a large number of tissue culture flasks would be required. Dealing with a large number of these flasks not only is very labor intensive but also tends to result in flask-to-flask variability. Additionally, handling multiple vessels increases the chance of contamination with external agents (i.e., bacterial). In this regard, the use of tissue culture flasks would not be appropriate for the expansion of a clinically relevant number of hMSCs. For the scalable expansion of hMSCs, there are many available bioreactors, including multi-layered cell factories, roller bottles, hollow fiber, packed beds, and stirred suspension bioreactors, to name a few. Each bioreactor has its own specific features (and benefits), and thus it is important to compare the different bioreactors and select the best one for the large-scale expansion production of quality-assured hMSCs. In this section, we will briefly review two main bioreactors, multi-layered cell factories and stirred suspension bioreactors (using microcarriers), which are currently used for the large-scale production of hMSCs.

### Multi-layered vessels

The multi-layered cell factory represents the simplest system for scaling up from monolayer culture as it has a geometry and substrate similar to those of a T-flask. It offers a large surface area for cell growth by layering stacks of ‘flask-units’ on top of each other. Typically, each cell factory ‘unit’ consists of 1 to 40 stacks that are connected together (i.e., Nunc Cell Factory, Thermo Fisher Scientific, Waltham, MA, USA; Corning CellSTACK; Corning, Corning, NY, USA), where extra layers can be added. And recent innovations, such as the Corning Hyperstack, have tripled the surface area per volume of traditional multilayer vessels and offer vessels containing 120 layers. This method of cell expansion has been used by many investigators to expand hMSCs [53–56]. In particular, Bartmann et al. [53] reported that the growth of hMSCs in cell factories was similar to that of T-225 flasks, and in order to obtain a clinical dose of hMSCs (>200 × 10^6^ cells), four to 10 four-layered cell factors were used. Owing to its easy implementation and scale-up achieved by simply increasing the size and number of layers, it has been used by companies in clinical trials as their main expansion technology [57]. However, this process is difficult to monitor and control throughout the culture period. Moreover, difficulties in achieving a uniform distribution of cells and in harvesting could result in increased culture heterogeneity and suboptimal yield of cells [58]. Thus, this system in its current form may not be ideal if higher cell doses are required for an application. Pall Life Sciences (Port Washington, NY, USA) has introduced a single-use bioreactor technology, Integrity Xpansion, that is a closed system containing up to 122,400 cm^2^ of growth area, and integrated temperature, dissolved oxygen, and pH control. Although this system has been shown to support expansion of MSCs, the potency of these cells has yet to be reported [59].

### Stirred suspension bioreactors

Stirred suspension bioreactors are relatively simple vessels that have a centrally located impeller, which agitates the contents of the vessel and provides relatively uniform conditions throughout the medium. The impeller speed is controlled by either a magnetic field generated by a stirrer placed under it or a top-driven motor. Currently, there are a number of stirred suspension bioreactors available at different volumes, such as the DASGIP Parallel Bioreactor system and Celligen (Eppendorf, Hauppauge, NY, USA), PADReactor (Pall Life Sciences), and MiniBio (Applikon Biotechnology, Delft, The Netherlands), to name a few. By means of stirred suspension bioreactors, a large number of cells can be expanded in one vessel, thereby avoiding vessel-to-vessel variability (i.e., as is the case with multiple T-flasks) and minimizing costs related to labor and consumables. Additionally, these bioreactors can be operated in a number of modes: batch (i.e., medium is not replaced), fed-batch (i.e., intermittent medium replacement), or perfusion (i.e., continuous medium replacement). Operating the bioreactors in a fed-batch or perfusion mode ensures that key nutrients are replenished and metabolic waste products (i.e., lactate and ammonium) are kept at safe levels. Furthermore, these bioreactors can be equipped with computer-controlled online-monitoring instruments that ensure tight control of process variables such as pH, temperature, and dissolved oxygen concentration. Moreover, single-use, closed bioreactors (e.g., Cultibag STR; Sartorius AG, Göttingen, Germany) are available, enabling GMP production of cells in a class C or D room and not in a class A cabinet or B room [60]. Based on these advantages, stirred suspension bioreactors have been used for the culture of stem cells, which grow as tissue aggregates or adherent cells using microcarriers.

Used in suspension culture, microcarriers are small beads that have a diameter of between 100 and 300 microns and provide a large surface-to-volume area for anchorage-dependent cells to attach and grow. They can be easily maintained in suspension in liquid medium and provide a high surface area-to-volume ratio (i.e., microporous microcarriers can provide a ratio of 30 cm^2^/cm^3^ medium at a bead loading of 10 g/l (Cytodex 3 microcarriers; GE Healthcare, Little Chalfont, UK), whereas T-flasks have a smaller ratio of 3 cm^2^/cm^3^ medium), which allows much higher cell yields to be achieved in suspension culture. These microcarriers are typically made of various materials, including collagen, dextran, and glass, which have varying surface properties that affect cell growth kinetics and phenotype.

A number of investigators have shown that MSCs derived from multiple sources, such as the bone marrow, placenta, and ear, could be expanded on microcarriers [61–63]. When this technology was first developed, the cell yield was low and variable compared with those of expanding cells in static culture flasks. Therefore, further efforts were required to optimize this culture system before it could be considered comparable to that of static culture flasks. Here, we will provide a brief overview of three main variables: (1) microcarrier selection, (2) microcarrier-loading/cell-seeding density, and (3) medium composition. For a further review, see [64]. Additionally, we will discuss the design considerations for scaling up suspension bioreactors.

#### Microcarrier selection

The selection of an appropriate microcarrier is important as it can impact the growth kinetics as well as the phenotype of the expanded cells. Moreover, microcarrier screening should be done in the same culture system as will be used for their large-scale implementation, in order to incorporate the influence of the culture environment on the performance of the microcarriers. Microporous microcarriers have been investigated for many years and can have different surface properties and coatings, which influence cell attachment and subsequent cell expansion. Also, the source and isolation method of hMSCs can affect their subsequent expansion on microcarriers, and thus it is important to identify one that works specifically for a given process. However, in general, it has been shown for hMSCs that cell-adhesive coatings (i.e., collagen) can promote attachment and proliferation of fastidious cells [65].

It has also been reported that macroporous and biodegradable microcarriers have been evaluated for the growth of hMSCs [66–68]. These microcarriers allow cells to grow internally and therefore are protected from the hydrodynamic shear present in the stirred bioreactors. Additionally, by using biodegradable microcarriers, hMSC recovery may be higher or the hMSCs and microcarriers could be transplanted in vivo without their separation. This may be beneficial if the therapeutic effect is intended to be localized at the site of administration. However, as in the development of serum-free media, it is also important to evaluate the use of animal component-free microcarriers. Three groups thus far have published results on the use of xeno-free microcarriers, which have shown the ability to support the growth of hMSCs [69–71].

#### Microcarrier-loading and cell-seeding density

Microcarrier density and cell-to-bead (microcarrier) ratio are well known variables that affect not only the initial cell attachment efficiency but also the level of culture compactness. Cell attachment to microcarriers follows a Poisson distribution [72], in which for a cell-to-bead inoculation ratio of 1, 2, 3, and 4 cells per bead, the theoretical probabilities of unoccupied microcarriers are 0.365, 0.135, 0.05, and 0.018, respectively. Additionally, these probabilities are likely to be increased under nonoptimal inoculation conditions (e.g., inhibitory medium components, suboptimal microcarrier type, cellular damage, or adverse pH). Therefore, it is important to inoculate cells at a sufficiently high cell-to-bead ratio in order to achieve a good distribution in which each bead is occupied by at least one viable cell. For hMSC culture, cell-to-bead ratios of between 3 and 5 cells per bead and microcarrier densities of between 1 and 4 g (dry weight) per liter have been used. Based on these typical cell inoculation and microcarrier densities (i.e., 2 g/l), most researchers have achieved a final hMSC concentration in the range of 1–4 × 10^5^ cells/ml [66, 70, 71]. In contrast, other mammalian cells used in industrial microcarrier culture achieve a final cell density of around 2 × 10^6^ cells/ml [73]. The large difference in final cell culture concentrations may be attributed to suboptimal culture conditions, including microcarrier-loading and cell-seeding densities. Therefore, to increase the final hMSC cell concentration, higher microcarrier densities may be used. However, with higher-density cultures, it may be necessary to increase medium oxygenation (i.e., sparging) and establish more frequent medium feedings to supply adequate oxygen and nutrients.

#### Medium composition

**Fig. 1:**
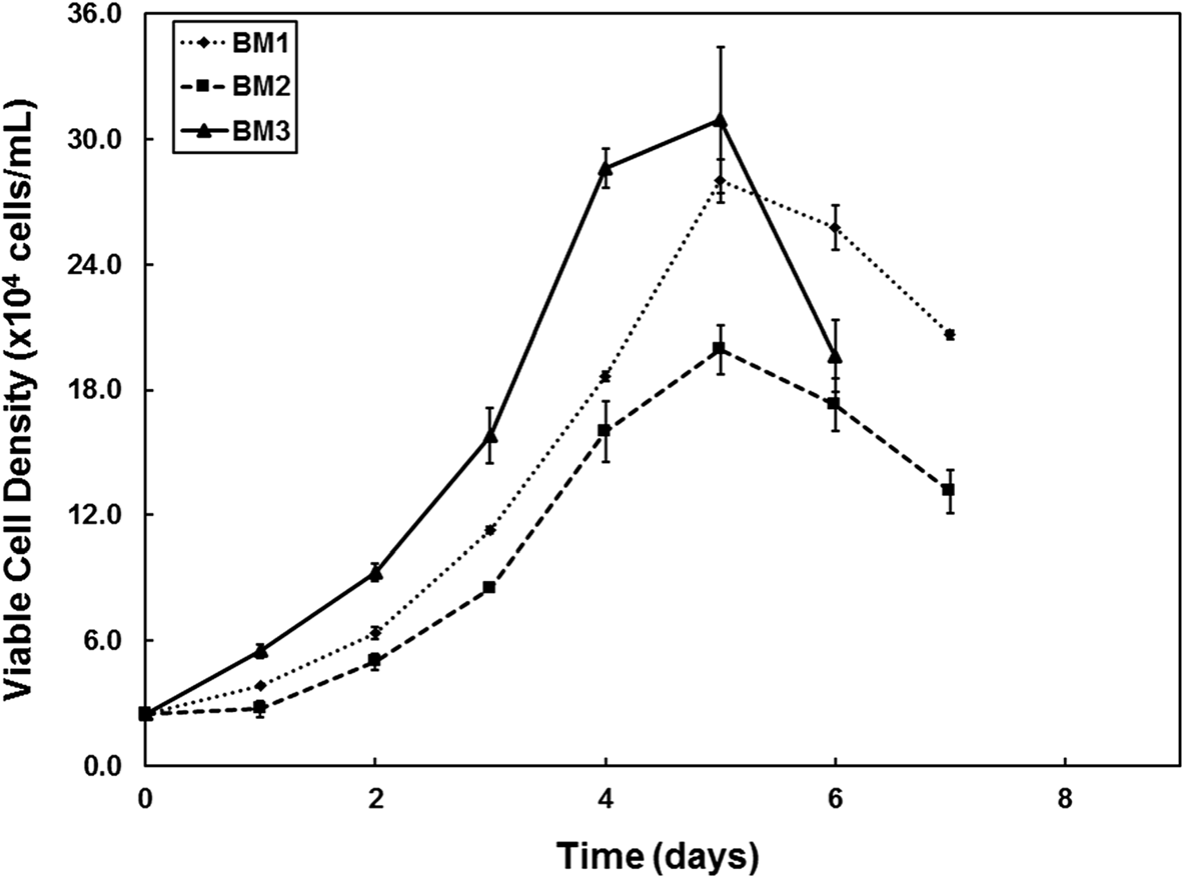
Expansion of bone marrow-derived human mesenchymal stem cells (BM-hMSCs) on Cytodex 3 microcarriers in serum-free PPRF-msc6 medium in 125-ml stirred suspension bioreactors [84]. hMSCs isolated in PPRF-msc6 were thawed and expanded for two passages in PPRF-msc6 and then inoculated at 2.4 × 10^4^ cells/ml in stirred suspension bioreactors containing 2.0 g/l of Cytodex 3 microcarriers. We observed variable cell growth kinetics between different BM donors (BM1, BM2, and BM3). This indicates that although this bioreactor system was optimized for the growth of one hMSC donor, inter-donor differences influence the growth kinetics of hMSCs in stirred suspension bioreactors. Error bars represent the observed range, n = 2

In conventional serum-based media, hMSCs in microcarrier culture exhibit a prolonged lag phase and low growth rate [66, 72, 74]. Minimizing the lag phase and maximizing the rate and length of the exponential growth phase are requirements in designing a good bioprocess. We have recently published results showing good expansion of hMSCs on Cytodex 3 microcarriers, in a serum-free medium formulation (PPRF-msc6), in 125-ml suspension bioreactors [64]. Compared with hMSCs expanded in 10 % FBS DMEM, hMSCs in PPRF-msc6 had a significantly shorter lag phase and reached a higher cell density at an earlier time point (4.38 ± 0.23 × 10^5^ cells/ml on day 6). Eibes et al. had also reported that using a low-serum medium significantly enhanced the expansion of hMSCs compared with 10 % FBS DMEM [66]. This has also been observed by researchers using other serum-free media to expand hMSCs in microcarrier culture [70, 71]. However, we also observed that different hMSC donors (BM1, BM2, and BM3) had variable growth kinetics in our 125-ml suspension bioreactors (Fig. 1) but that in concurrent static T-flasks, the growth kinetics of the cells were comparable. This may be due to optimizing our microcarrier bioprocess by using one hMSC donor (BM3) while our other donors might require different culture parameters (e.g., microcarrier type and cell-seeding density). This would explain why the maximum cell density achieved is higher for BM3 cells compared with the other two hMSC cell lines (Fig. 1). Therefore, although microcarrier technology is an attractive option for the production of clinically relevant hMSCs, a number of variables will be need to be optimized and standardized for the development of a consistent, high-performance bioprocess.

### Design considerations for scale-up of suspension bioreactors

To scale-up bioreactor cultures, two key variables need to be taken into account: (a) oxygen supply and (b) hydrodynamic shear in the liquid medium. The specific oxygen consumption rate of exponentially growing mammalian cells has been reported to be between 1.7 × 10^−17^ and 17.0 × 10^−17^ mol O_2_/cell∙s [73]. If cells use oxygen faster than it is being supplied to a bioreactor, then the dissolved oxygen level will decrease to a point where the culture may not support cell growth. Gilbertson showed that with surface aeration, for the culture of mouse neural stem cells, the mass transfer of oxygen from the headspace to the bulk medium would support the oxygen demands of cells at a density of 1 × 10^6^ cells/ml and would not be limiting up to 1.0 l of culture in a cylindrical-shaped bioreactor [75]. Given current hMSC growth kinetics, surface aeration would be adequate for scaling-up hMSC suspension bioreactor bioprocesses to 1.0 l. Further studies are needed to address the oxygen limitation issue at higher scales.

Hydrodynamic shear is another important characteristic to consider. In stirred suspension bioreactors, the agitation rate of the impeller governs the hydrodynamic shear within the vessel, and as the agitation increases the hydrodynamic shear rate increases. If the agitation rate is too low, the culture may not be well mixed, causing problems such as a significant aggregation of cells and microcarriers and nonhomogeneous culture environment. The uncontrolled aggregation may cause limited transfer of oxygen and nutrients to cells inside large aggregates. However, if the agitation rate is too high, this can be detrimental if it causes excessive cell damage. To estimate the hydrodynamic shear, the Kolmogorov’s theory of turbulent eddies [76] and the Nagata correlation [77] have typically been used in order to maintain the same maximum shear rate. However, this calculation does not take into account the flow regime present in the vessel and these values differ between different bioreactor configurations that may impact the growth of the cells. Consequently, it has also been suggested that suspension experiments and computational fluid dynamics studies combined with particle image velocimetry measurements be used to determine optimal scale-up operating parameters [78].

## Cell-based (hMSC) or cell-free therapy (hMSC secretome)?

As discussed, when therapeutically viable hMSCs are generated, it is important to consider the effect that the bioprocess has on the cell yield and cell properties. Moreover, it is important to consider the downstream process and, in particular, how these cells might be transplanted into patients in hospitals. Currently, the majority of hMSC clinical trials are administering hMSCs as freshly thawed cells [79–81]. This is because cells are produced in one location, tested for sterility, and then shipped to transplantation centers where they may not be administered immediately. Therefore, it is cost-effective to freeze the cells and thaw them only once they are needed. However, this may be unwise as all pre-clinical studies of hMSCs in disease models usually involve transfusion/transplantation of live MSCs harvested during their log phase of growth.

Recently, it was shown that the therapeutic properties of hMSCs are impaired by this freeze–thaw [80, 82]. Moreover, if the cells were thawed and cultured in vitro, the hMSCs reverted back to their noncryopreserved phenotype and recovered their therapeutic properties [79, 80]. However, this may not be possible in a hospital setting where specialized equipment is required and specifically trained personnel who can generate the hMSCs are required for each patient disease intervention. In this regard, the production of the hMSC secretome (i.e., the CM: the medium containing the hMSC-secreted factors but is cell-free) may present a better avenue for the clinical application of hMSCs as it has been shown that this medium can be injected in vivo for clinical benefit [34, 44, 48]. Additionally, it has been shown that by altering the culture environment, the therapeutic properties of hMSCs and their secreted products can be modulated [33, 34, 44, 48].

**Fig. 2:**
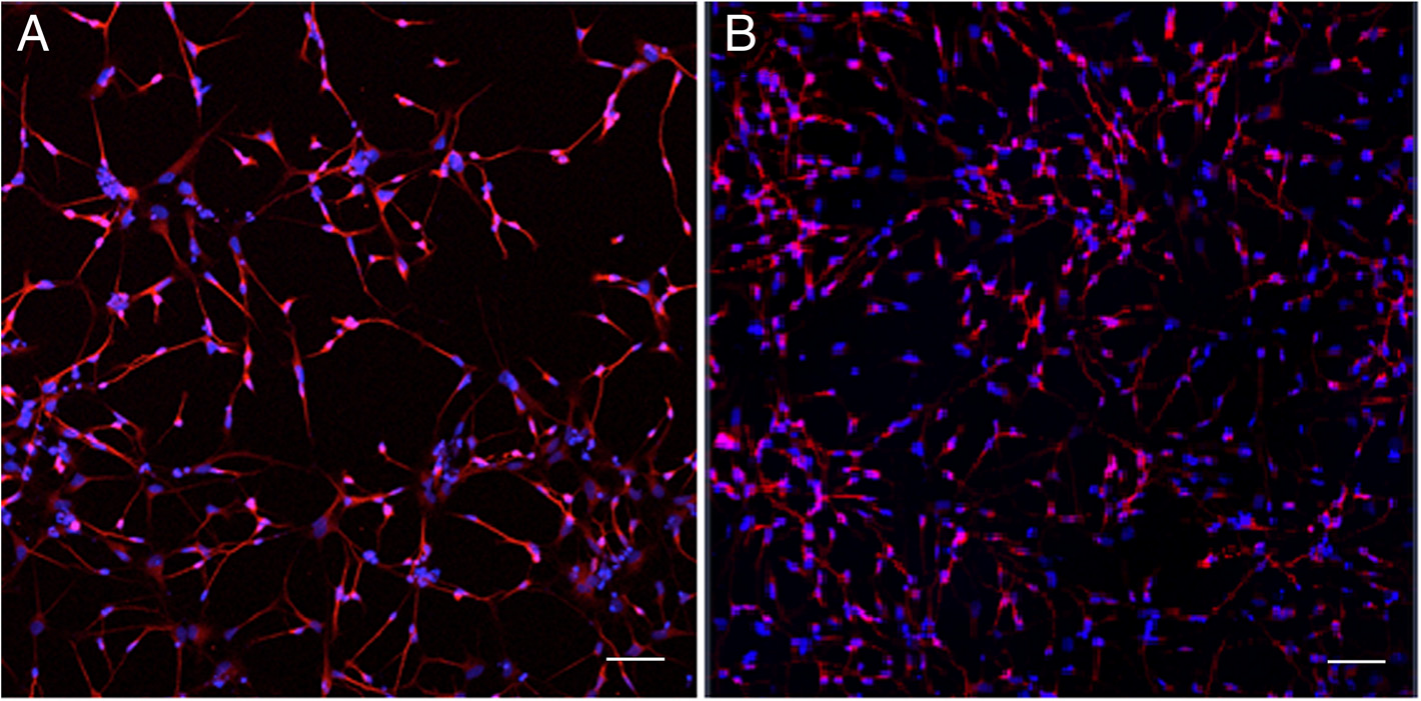
Differentiation of human telencephalon-derived neural stem/progenitor cells (hNPCs) in conditioned medium collected from bone marrow-derived human mesenchymal stem cells (BM-hMSCs) expanded in either (**a**) static culture in fetal bovine serum (FBS)-based medium (T-flasks) or (**b** )500-ml computer-controlled suspension bioreactors in PPRF-msc6 medium [84]. Shown are cells expressing MAP2 (*red*) and TO-PRO-3 (*blue*). Conditioned medium collected from hMSCs expanded in PPRF-msc6/bioreactor resulted in a higher differentiation of hNPCs to MAP2^+^ neurons. Additionally, hNPC survival was higher in the PPRF-msc6/bioreactor-expanded hMSC conditioned medium. In contrast, hNPCs incubated in the FBS/static-expanded hMSC conditioned medium had a lower survival and differentiation into MAP2^+^ neurons. This indicates that the conditioned medium collected from our PPRF-msc6/bioreactor conditions contained factors that promoted the survival and differentiation of hNPCs into neurons. However, the FBS/static conditioned medium was less effective in causing hMSCs to secrete these factors. Scale bars: 50 μm

We recently observed that we can enhance the neurotrophic properties of hMSCs by using PPRF-msc6 medium and our computer-controlled stirred suspension bioreactors compared with conventional culture in static culture flasks and 10 % FBS DMEM (Fig. 2). Specifically, using the Kolmogorov’s theory of turbulent eddies and the Nagata correlation, we scaled-up our hMSC cultures from 125-ml suspension bioreactors to 500-ml computer-controlled bioreactors on the basis of maintaining the same maximum shear. hMSCs were inoculated at 4444 cells/cm^2^ either into static culture flasks containing 10 % FBS DMEM or into computer-controlled stirred suspension bioreactors (DASGIP) containing 500 ml of PPRF-msc6 medium with 2 g/l of Cytodex 3 microcarriers. The cells were expanded for 72 hours, after which the cultures were incubated with an equivalent amount of Neurobasal-A medium (Life Technologies, Carlsbad, CA, USA) for 24 hours at the same culture process parameters. This medium (herein called the CM) was collected after 24 hours. When the CM was incubated with human neural precursor cells (hNPCs) (containing both stem and progenitor cells; see discussion in [83]) for 7 days, the hNPC survival was significantly higher in the PPRF-msc6/bioreactor CM compared with the FBS/static-expanded CM. Additionally, differentiation of hNPCs into MAP2^+^ neurons was significantly higher for hNPCs incubated with the PPRF-msc6/bioreactor CM compared with the FBS/static-expanded CM. These data suggest that the use of computer-controlled stirred suspension bioreactors with PPRF-msc6 can enhance the neurotrophic potential of hMSCs. Therefore, by altering the hMSC culture mode, we can generate novel trophic cocktails (i.e., the CM) that could be produced centrally at one (or multiple locations) in accordance with GMP methods and then concentrated, frozen, and shipped in ready-to-use packages. This would negate the issue of setting up specialized cell culture facilities within the hospital and the hiring of cell culture technicians and the hassle of planning patient interventions to coincide with optimal hMSC harvesting during the log phase of growth and would allow standardization of hMSC treatments.

## Conclusions

hMSCs are currently being evaluated as a stem cell treatment for a number of diseases and have been shown to be safe in clinical trials. They are able to elicit their therapeutic benefits through the secretion of bioactive molecules that modulate the in vivo environment and promote tissue repair/regeneration. However, current methods to generate hMSCs suffer from variable culture conditions because of ill-defined medium, heterogeneous culture environment, and limited growth surface area per culture. Additionally, the in vitro culture environment has been shown to modulate and influence the therapeutic ability of hMSCs and their secretome. Thus, to meet the current and future demand of clinically relevant numbers of hMSCs, it is necessary to develop a bioprocess that is well defined, scalable, and under good process control which can be operated in accordance with GMP. To this end, much research has gone into investigating and optimizing a number of ‘variables’ in the hMSC culture environment. This research includes (1) the development of serum-free media, (2) modification of the traditional culture environment, and (3) development of scalable and controllable culture systems.

## Note

This article is part of a thematic series *Mesenchymal stem/stromal cells—an update*. Other articles in this series can be found at http://www.biomedcentral.com/series/mesenchymal.

## Abbreviations

BM-hMSC: Bone marrow-derived human mesenchymal stem cell
CM: Conditioned medium
DMEM: Dulbecco’s modified Eagle’s medium
FBS: Fetal bovine serum
GMP: Good Manufacturing Practice
HGF: Hepatocyte growth factor
hMSC: Human mesenchymal stem cell
hNPC: Human neural precursor cell
hPL: Human platelet lysate
MNC: Mononuclear cell
PD: Population doubling
TBI: Traumatic brain injury
TNF-α: Tumor necrosis factor-alpha
VEGF: Vascular endothelial growth factor
